# Addressing Emergency Department Care for Patients Experiencing Incarceration: A Narrative Review

**DOI:** 10.5811/westjem.59057

**Published:** 2023-06-28

**Authors:** Rachel E. Armstrong, Kristopher A. Hendershot, Naomi P. Newton, Patricia D. Panakos

**Affiliations:** * University of Miami, Miami, Florida; † Jackson Health System, Miami, Florida

## Abstract

Patients experiencing incarceration face a multitude of healthcare disparities. These patients are disproportionately affected by a variety of chronic medical conditions. Patients who are incarcerated often remain shackled throughout their hospital course, experience bias from members of the healthcare team, and have many barriers to privacy given the omnipresence of corrections officers. Despite this, many physicians report little formal training on caring for this unique patient population. In this narrative review, we examine the current literature on patients who are incarcerated, especially as it pertains to their care in the emergency department (ED).We also propose solutions to address these barriers to care in the ED setting.

## INTRODUCTION

The United States has over 1.6 million incarcerated people.^1^ This population has been historically medically underserved and faces a variety of healthcare disparities. Individuals who are incarcerated are more likely than the general population to have medical conditions such as diabetes, hypertension, HIV, hepatitis C, and tuberculosis.^2^,^3^ The often substandard living conditions in jails and prisons also negatively impact incarcerated patients’ health. For example, the morbidity and mortality from COVID-19 was significantly higher in prisons than in the general public.^4^,^5^ While incarceration sometimes connects individuals who have not had previous access to care with continuity of care and medication for chronic conditions, many individuals are still unable to access adequate treatment while incarcerated.^6^,^7^ For example, cancer patients report inadequate access to pain medications, patients face barriers to acute surgical care, and pregnant patients report inadequate prenatal care.^8^,^9^,^10^ Even when patients are able to access care while incarcerated, they often face immense barriers to healthcare once released.^2^,^3^

In addition to the disparities noted above, incarceration is associated with mental illness and early mortality. When compared to non-incarcerated people, those who are incarcerated have higher rates of major depression, bipolar disorder, and schizophrenia.^11^–^16^ Furthermore, incarceration itself may predispose individuals to mental illness, as experiencing incarceration is a risk factor for developing a first psychotic episode.^17^ Substance use disorders (SUD) are more prevalent in the incarcerated population than the general population.^18^ Many correctional facilities do not provide adequate treatment for SUD, which can lead to situations of life-threatening withdrawal in individuals with benzodiazepine and alcohol use disorder.^19^,^20^ Individuals with opioid use disorder have a markedly increased risk of opioid overdose after release, especially if they are not started on medication-assisted treatment while incarcerated.^21^–^24^ With regard to mortality, studies have shown that people who have been incarcerated have an increased risk of death at a younger age when compared to the general population.^25^,^26^ This risk of premature death in incarcerated people disproportionately affects Black populations when compared to other demographic groups.^27^,^28^

It is impossible to discuss the disparities faced by incarcerated patients without recognizing that the criminal justice system is one based on racial oppression.^29^ Black Americans are incarcerated, wrongfully convicted, and stopped and searched by police at disproportionately higher rates than White Americans.^30^–^33^ The history of policing is also rooted in systemic racism. In the 18^th^ and 19^th^ centuries, groups called “slave patrols” would search for and detain enslaved people who escaped; these groups are considered the basis of “modern-day policing.”^34^,^35^ When formal police departments were established in the early 20^th^ century, these organizations served a large role in enforcing Jim Crow laws (laws in the South that institutionalized racial segregation, such as requiring separate water fountains for Black and White people).^34^ In the late 20^th^ century, the systemic criminalization of recreational drug use from President Ronald Reagan’s “War on Drugs” and President Bill Clinton’s Violent Crime Control and Law Enforcement Act disproportionately targeted Black and Latino Americans.^36^,^37^ These are some examples, but by no means an exhaustive list, of how systemic racism is linked to the criminal justice system in the US.

While this review focuses on patients who are incarcerated, patients present to the emergency department (ED) in various types of custody. Often patients are brought to the ED after they are arrested but before they are convicted of a crime so that emergent medical concerns can be addressed prior to booking. Some patients are brought in while detained by US Immigration and Customs Enforcement officers. Patients may also present to the ED during the pre-trial period or post-conviction from jail or prison. Patients from both jails and prisons experience barriers to healthcare, but there are great discrepancies in the care provided at jails, based on the variation in a jail’s size and resources and given that people typically spend less time in jails than in prisons.^6^,^38^,^39^ Additionally, smaller jails may contract out most of their medical services, and jails are subject to less regulatory healthcare oversight than prisons.^39^ While we will focus on the care of individuals who are incarcerated, many of the principles outlined in this article are applicable to patients in various types of custody.

Physicians employed by jails and prisons face an ethical dilemma termed “dual loyalty,” meaning the conflict in interest between caring for their patient and catering to the demands of the prison administration.^40^ Sometimes, physicians are asked to perform tasks that go against their role as healers, ie, to perform drug tests without a patient’s consent, to withhold an expensive medical treatment despite it being the standard of care, and to perform medical exams for the purpose of “certify[ing] that prisoners are fit for imprisonment.”^40^ Similarly, emergency physicians must be aware of the conflicts of interest that arise when caring for patients who are incarcerated, such as cases when they are asked to “medically clear” a patient prior to booking or perform tests or exams that are not clinically indicated.

Penal harm refers to any “planned governmental act whereby a citizen is harmed” for punitive reasons; the harm is considered “justifiable precisely because it is an offender who is suffering.”^41^,^42^ Although the Eighth Amendment of the US Constitution broadly “prohibits cruel and unusual punishment,” it was not until the 1976 Supreme Court ruling in the case of *Estelle v Gamble* that penal harm in the context of medical care was explicitly deemed unconstitutional.^43^ The *Estelle v Gamble* ruling, which centered on “the deliberate indifference of the medical needs of prisoners,” set a clear precedent for the rights of incarcerated patients to accessible medical care (including inpatient and specialist care).^43^,^44^ Failure to provide incarcerated patients with the same “standard of care” as non-incarcerated patients has henceforth been considered a violation of the Eighth Amendment.^44^ However, in practice, upholding the healthcare rights of incarcerated patients is more challenging to enforce.^45^

In this narrative review, we will identify several barriers to maintaining the standard of care for incarcerated patients in the ED. We hope to increase awareness of these disparities and propose solutions to better address them in clinical practice.^34^,^46^

## BARRIERS TO CARE

Through our review of the existing literature, we identified multiple barriers to treating incarcerated patients in the ED: biased care from physicians; presence of law enforcement; and use of physical restraints.

### Bias

Members of the healthcare team, including physicians and nurses, often have their own preconceived notions about incarcerated patients that ultimately affect patient care. A survey study of formerly incarcerated individuals found that many patients have experienced discrimination based on their criminal record.^47^ Many patients also reported discrimination in the healthcare setting due to their race and ethnicity.^47^ This survey study also found that formerly incarcerated individuals with increased ED utilization reported a higher rate of discrimination from healthcare professionals.^47^

However, many physicians already recognize the disparities in the care of patients who are incarcerated. One recent qualitative study by Douglas et al acknowledges the need to optimize quality of care for incarcerated patients.^48^ In this paper, surgical residents were surveyed about their encounters with law enforcement while caring for patients experiencing incarceration. The surgical residents noted many challenges when caring for these patients, including barriers to adequate follow-up care and the designated holding areas for incarcerated patients that may contribute to “substandard care” or decreased monitoring of critically ill patients.^48^

Bias has been reported among many members of the healthcare team. The study “Caring for Hospitalized Incarcerated Patients: Physician and Nurse Experience” by Brooks et al examined physician and nurse experiences when caring for hospitalized incarcerated patients using open and closed-ended survey questions.^49^ A majority of physicians believed patients who were incarcerated received less frequent non-medical interventions (defined as “social work support, physical therapy visits, nutrition consults”) during hospitalization than other patients.^49^ Over 30% of physicians believed that these patients received “fewer diagnostic tests” and “fewer medical interventions” than other patients.^49^

### Patient Privacy

There are also many limits to patient privacy when caring for patients who are incarcerated in the ED. The presence of corrections officers who accompany these patients to the ED often leads to protected health information (PHI) being shared if members of the healthcare team do not ask officers to step away during the history and physical.^43^,^50^

In the survey study by Brooks et al, a higher percentage of nurses when compared to physicians reported that they kept law enforcement in the exam rooms when performing their histories and physicals.^49^ Still, 35% of physicians reported not asking corrections officers to leave during patient encounters, and over 50% of the physicians reported not asking for shackles to be removed during their histories and physicals.^49^ In the survey, physicians also identified a lack of formal training in their medical education when caring for this group of patients.^49^

Many surgical residents in the Douglas et al study recognized incarcerated patients’ barriers to privacy. The majority of residents in this study reported witnessing incidents when law enforcement officers would question patients during trauma assessments, at times disrupting the primary and secondary survey and impinging on patient privacy.^48^ In addition, many residents reported instances when an “armed guard was present in the operating room” during a surgical procedure.^48^ One resident reported an instance when an officer requested an ethanol level on a patient, even though this test was not pertinent to the patient’s care at that time.^48^

The literature also describes instances when law enforcement, namely police officers, have requested invasive body searches and tests on patients, although these tests were not clinically indicated.^51^,^52^ In a survey study, emergency physicians reported breaches of patient privacy in the presence of law enforcement, including instances when officers solicited PHI.^53^ Physicians reported being uncertain of the exact role and limitations of law enforcement in their workplace.^53^

While physicians should always strive to maintain patient privacy, there are circumstances in which aspects of patient care may need to be disclosed to law enforcement. For example, PHI may need to be disclosed if a patient requires specific treatment or follow-up care in the outpatient setting. Given the delays that can occur in the care of incarcerated patients, instructions may need to be explicitly written or discussed with law enforcement to ensure appropriate care occurs after discharge from the ED.^54^ However, physicians should always attempt to obtain approval from patients prior to sharing their PHI. There are also instances where incarcerated patients may exhibit violent behavior that poses a safety threat to themselves or to ED staff. In these instances, it is appropriate to interview patients in the presence of law enforcement.

### Physical Restraints

Physicians are taught to use physical restraints with caution and only when absolutely necessary. Physical hospital restraints, which are often applied to protect patient safety, are associated with numerous complications. For example, there is a statistically significant increased incidence of pulmonary embolism and deep vein thrombosis in patients who are physically restrained.^55^,^56^ Furthermore, restraints are associated with delirium, emotional distress, rhabdomyolysis, injury, and even death when improperly used.^57^–^59^ Indeed, both the American College of Emergency Physicians (ACEP) and The Joint Commission have published standards on the criteria necessary to justify restraint use and minimize harm associated with restraints.^59^–^61^

Despite the caution advised when using physical restraints, patients who are incarcerated often arrive to the ED in shackles and remain in shackles throughout the course of their ED stay. Some surgery residents have even reported caring for patients who are shackled to the bed while intubated and sedated.^48^ There are some policies in place to limit the use of shackles in clinical settings. Recognizing the risks of physical restraints in pregnancy, many states have mandated against physical restraints for patients who are incarcerated in the perinatal period.^62^ Federal policies have also been enacted to restrict use of physical restraints in pregnancy, except when considered necessary for safety reasons.^62^

There is a dearth of protections for patients who are not pregnant. Non-pregnant, incarcerated patients often remain shackled throughout their hospital stay; this includes those who are terminally ill and those who are intubated and sedated.^63^–^65^ There is little data to support the medical rationale for shackling and, indeed, its use is mainly determined by federal and local policy to be a requirement during transport.^63^,^64^,^66^–^68^ A discussion on ways to address shackling in the ED is included below.

## STRATEGIES TO IMPROVE CARE

In this section, we propose several strategies to improve the quality of ED care for patients who are incarcerated. These suggestions are not exhaustive; much more research is required to further investigate the many disparities these patients face.

### Bias

Hofmeister and Soprych discuss the importance of including formal teaching on treating incarcerated patients in medical curricula.^69^ The authors discuss how the use of workshops on implicit biases can be incorporated into resident medical education. The workshop they performed allowed resident physicians to self-reflect on their biases and better recognize the disparities that specifically affect incarcerated patients.^69^ There should be increased curriculum development in medical education that focuses on addressing the biases faced by patients who are incarcerated.

### Privacy

The US Department of Health and Human Services outlines PHI protections for patients who are incarcerated. Sharing of PHI is only permitted in a few distinct circumstances, such as when healthcare clinicians are responding to a request for “PHI [that] is needed to provide health care” to the patient, or when the PHI is necessary to protect the health/safety of the individual or people around them.^70^ As one can imagine, information may be inadvertently divulged to corrections officers if the emergency physician (EP), nurse practitioner (NP), or physician assistant (PA) conducts the history and physical with corrections officers in the room.^43^ A toolkit for protecting patient privacy created by the Working Group on Policing and Patient Rights of the Georgetown University Health Justice Alliance recommends that EPs, NPs, and PAs ask officers to step out of “earshot” to protect PHI and “prevent accidental disclosures.”^50^ EPs, NPs, and PAs should also obtain patient consent prior to lab tests and procedures.^50^ The “Medical Provider Toolkit” and ACEP also note that while law enforcement personnel may even provide warrants for specific tests and exams such as body cavity searches, EPs, NPs, and PAs can refuse if they are not clinically indicated or are not in the patient’s best interest.^50^,^71^

### Physical Restraints

Just as certain federal and state policies advocate for limiting shackle use in pregnant patients, so too should there be a greater emphasis placed on the removal of shackles on patients who are not pregnant. When interviewed, many physicians and nurses reported not requesting that shackles be removed.^49^ As mentioned above, there is little data to support the use of shackles, and many of the rules and regulations regarding shackle use focus on transportation. Barriers to shackle removal are often due to knowledge deficits and unclear institutional guidelines surrounding shackling. EPs, NPs, and PAs should recognize the harms associated with shackles and request their removal whenever possible, as these are often not medically necessary.^50^,^64^ Indeed, the International Association for Healthcare Security & Safety states that it is the responsibility of the physician and other members of the healthcare team to “assess the safety of continued use of restraint.”^72^ In addition, it is the duty of the corrections officers, and not the medical team, to ensure the patient’s security.^64^ Given that there are often unclear guidelines surrounding shackles and non-medical restraints, hospitals should also set forth their own guidelines to uphold the principle of medical non-maleficence in all treatment areas including the ED.^64^

To minimize harm, physicians should avoid and advocate against the shackling of patients in the prone position. This type of restraint confers an even greater risk of complications and has been linked to cardiopulmonary arrest, especially in agitated patients.^73^ Controversy remains as to whether this is secondary to positional asphyxia; Steinberg provided a review of the current literature detailing how the cause of sudden death in prone restraint is “multifactorial,” resulting from “reduced cardiac output,” metabolic acidosis, and impaired ventilation.^73^ While the Joint Commission does not explicitly prohibit prone restraint, hospitals are required to report any deaths that occur while a patient is restrained.^74^,^75^ Since prone restraint has been identified as a contributor to death in subjects who are agitated, many institutions have created policies against its use.^76^,^77^

### Advocacy

We encourage EPs to advocate for change in the carceral system and in their own institutions to improve the healthcare of patients who are incarcerated. Issues of inadequate living conditions in prisons, prison crowding, and discrimination outside the hospital have been well documented.^78^–^81^ Given the health implications of these issues, physicians should recognize and advocate for better living situations for these vulnerable patients.^82^ Conditions for patients who are incarcerated are sometimes inadequate in the hospital. Some hospital EDs have separate holding areas for patients who are incarcerated. The quality of care in these ED holding areas could be improved by increasing the staffing, resources, and attention to these sections.^48^ Physicians should advocate for better conditions for incarcerated patients, both within the ED and without.

### Continuity of Care

In addition to the reported substandard care that incarcerated patients receive while in the hospital setting, there are many barriers to appropriate medical care in correctional facilities. The article “Emergency Medical Care of Incarcerated Patients: Opportunities for Improvement and Cost Savings” by Martin et al is a chart review of incarcerated patients’ ED visits at a single institution.^54^ Patients reported barriers to care, such as difficulty accessing prescription medications for chronic conditions.^54^ In light of this, EPs, NPs, and PAs should ask patients who are incarcerated about their access to medications for chronic conditions and refill appropriate prescriptions prior to discharge. In addition, there are many documented cases of patients eventually presenting to the medical system with late-stage illnesses that could have been treated earlier if they had been previously identified.^79^ We encourage EPs, NPs, and PAs to refer patients to specialists and recommend clinic visits when appropriate.^79^

### Education

There is a lack of formal education surrounding care for incarcerated patients. In addition to bias workshops, the implementation of lectures, case-based discussions, and simulation cases can provide residents, attending physicians, NPs, and PAs with the tools necessary to care for this unique patient population. We developed and successfully implemented a simulation case for resident learners involving the presentation of a patient experiencing incarceration. This simulation aimed to expose learners to the issues unique to incarcerated patients as well as promote discussion on the removal of shackles during ED care, implicit biases, and protecting PHI. We are in the process of analyzing survey data from this simulation session, and results are forthcoming.

## CONCLUSION

Incarcerated patients are part of a vulnerable population that currently receives substandard care in many healthcare settings, including EDs. The biases held by members of the healthcare team, the presence of corrections officers, and pervasive use of restraints contribute to the numerous healthcare inequities. We have proposed strategies to improve the quality of care for this group of patients, recognizing that changes must be made on the physician level, throughout medical education, and institutionally.

*“Medicine should be viewed as social justice work in a world that is so sick and so riven by inequities.”* 83

– Dr. Paul Farmer.
